# Supplementary material for: Mutations in PCYT2 disrupt etherlipid biosynthesis and cause a complex hereditary spastic paraplegia
Source: Brain. 2019 Oct 22;142(11):3382–97. doi: 10.1093/brain/awz291 (PMC6821184; doi:10.1093/brain/awz291)
Supplement: awz291_Supplementary_Data [file awz291_supplementary_data.zip › awz291-Suppl_data/Supplementary_Data4.zip!/Supplementary information 4 - plasma lipidomics.zip!/Plasma lipidomics/Control vs Patient3a/Statistical plots and data/TG species/Volcano plot_alternative.pdf]

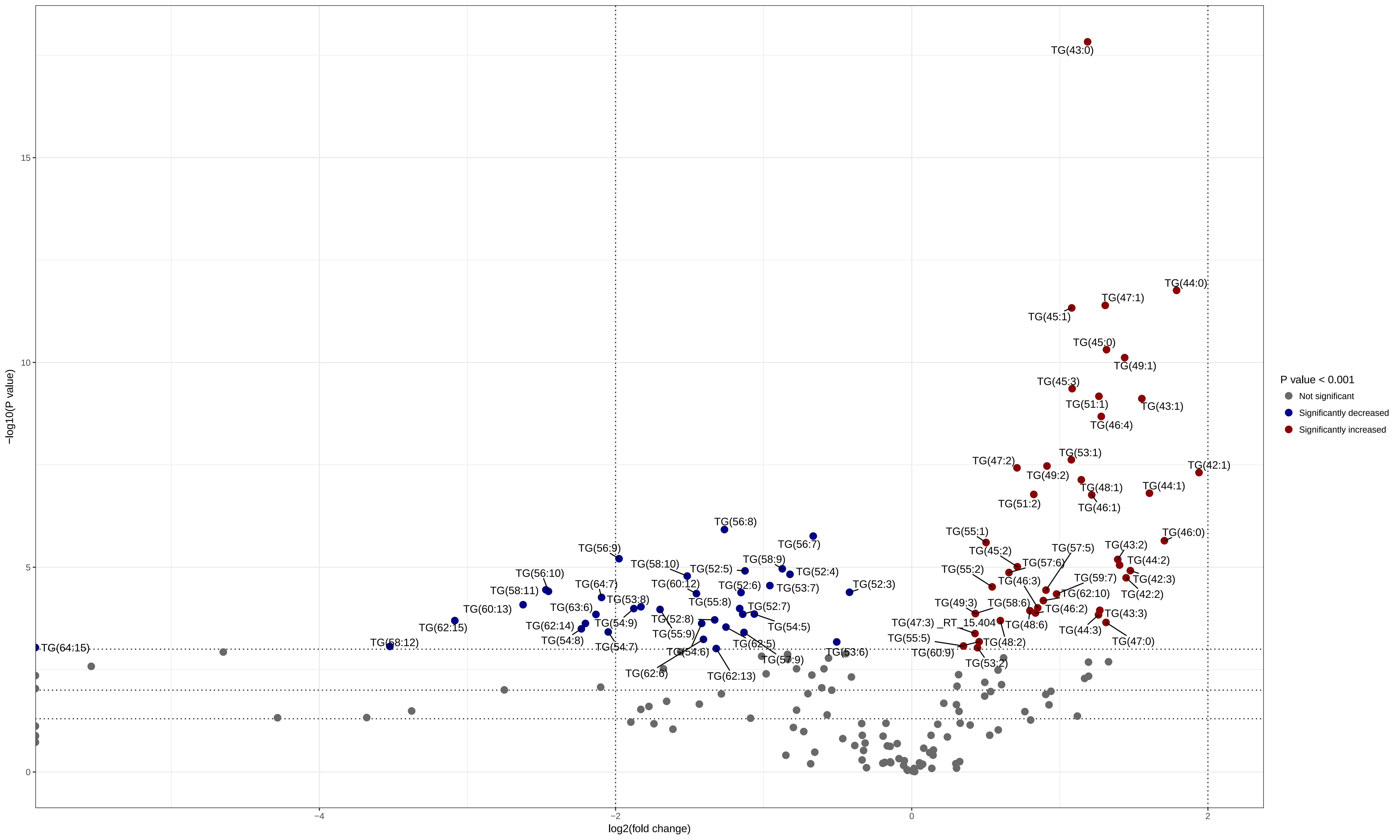
TG(43:0)
TG(44:0)
TG(47:1)
TG(45:1)
TG(45:0)
TG(49:1)
TG(45:3)
TG(51:1)
TG(43:1)
TG(46:4)
TG(53:1)
TG(47:2)
TG(49:2)
TG(42:1)
TG(48:1)
TG(44:1)
TG(51:2)
TG(46:1)
TG(56:8)
TG(55:1)
TG(46:0)
TG(56:7)
TG(56:9)
TG(57:5)
TG(43:2)
TG(45:2)
TG(44:2)
TG(58:9)
TG(57:6)
TG(58:10)
TG(52:5)
TG(52:4)
TG(55:2)
TG(59:7)
TG(42:3)
TG(56:10)
TG(64:7)
TG(60:12)
TG(52:6)
TG(53:7)
TG(52:3)
TG(46:3)
TG(62:10)
TG(42:2)
TG(58:11)
TG(53:8)
TG(55:8)
TG(49:3)
TG(58:6)
TG(60:13)
TG(63:6)
TG(52:7)
TG(46:2)
TG(43:3)
TG(52:8)
TG(54:9)
TG(47:3)_RT_15.404
TG(48:6)
TG(62:15)
TG(62:14)
TG(54:5)
TG(44:3)
TG(55:9)
TG(47:0)
TG(54:8)
TG(58:12)
TG(62:5)
TG(55:5)
TG(48:2)
TG(64:15)
TG(54:7)
TG(54:6)
TG(53:6)
TG(60:9)
TG(57:9)
TG(53:2)
TG(62:6)
TG(62:13)
15
10
5
0
−4
−2
0
2
−log10(P value)
log2(fold change)
P value < 0.001
Not significant
Significantly decreased
Significantly increased
